# *Mycoplasma bovis* arthritis and pneumonia in calves in Jordan: An emerging disease

**DOI:** 10.14202/vetworld.2018.1663-1668

**Published:** 2018-12-12

**Authors:** Wael M. Hananeh, Waleed M. Al Momani, Mustafa M. Ababneh, Sameeh M. Abutarbush

**Affiliations:** 1 Department of Veterinary Pathology and Public Health, Faculty of Veterinary Medicine, Jordan University of Science and Technology, P.O. Box, 3030, Irbid 22110, Jordan; 2 Department of Basic Medical Sciences, Faculty of Medicine, Yarmouk University, Irbid, Jordan; 3 Department of Basic Veterinary Medical Sciences, Faculty of Veterinary Medicine, Jordan University of Science and Technology, P.O. Box 3030, Irbid 22110, Jordan; 4 Department of Veterinary Clinical Sciences, Faculty of Veterinary Medicine, Jordan University of Science and Technology, Irbid 22110, Jordan

**Keywords:** arthritis, bovine, *Mycoplasma bovis*, pathology, pneumonia

## Abstract

**Aim:**

Clinical, microbiological, molecular, and pathological assays were undertaken to characterize an outbreak of increasingly reported signs of unresponsive arthritis and pneumonia of *Mycoplasma bovis* infection in young calves in Jordan.

**Materials and Methods:**

Clinical history of the affected bovine herd was investigated for the presence of respiratory and/or joint problems. Two calves with such history were clinically examined and necropsied. Representative tissues were sent for microbiological, molecular, and pathological examinations for *M. bovis* infection.

**Results:**

The outbreak started in a herd of 220 nursing calves, 2 months before the receiving of two calves for postmortem examination. Clinically, respiratory signs and infection of one or more joints dominated in the affected calves. The morbidity and case fatality rates were 27.27% and 61.7%, respectively. The left carpal joint was markedly swollen in both calves and exhibited necrofibrinous to granulomatous arthritis in varying degrees of severity. The anteroventral lung lobes in both calves were consistently affected and revealed multifocal to coalescing severe necrogranulomatous and fibrinopurulent bronchopneumonia. Microbiological and molecular findings confirmed the pathological examination. Furthermore, bovine viral diarrhea (BVD) was diagnosed in one calf by histopathology and polymerase chain reaction.

**Conclusion:**

This investigation reports the first outbreak of *M. bovis* infection in calves located in Jordan that could occur concurrently with BVD.

## Introduction

*Mycoplasma bovis* was first recognized in 1961 when Hale *et al*. [1] isolated the mycoplasma from cattle with severe mastitis in the USA. Since then, mycoplasma infection has been reported in cattle throughout the world [2]. *M. bovis* can cause a variety of diseases including pneumonia, arthritis, tenosynovitis, mastitis, keratoconjunctivitis, and otitis in cattle. Among other causative agents for bovine respiratory disease (BRD) in feedlot cattle, *M. bovis* is a commonly diagnosed and reported etiological agent [3]. Furthermore, *M. bovis* has caused severe outbreaks in feedlot bison [4-6]. Those outbreaks were characterized by severe chronic pneumonia, arthritis, and polyarthritis with high morbidity.

*Mycoplasma* spp. are important etiological agents of the BRD complex. They belong to the Mollicutes class, of which *Mycoplasma mycoides* subsp. mycoides small colony, *M. bovis*, and *Myrmecia dispar* are the most important species related to BRD [7,8]. Mycoplasmas inhabit mucosal surfaces of the respiratory, urogenital, and gastrointestinal tracts, as well as the eyes and mammary glands [6]. Their relationship with the host varies from primary or opportunistic pathogens to commensals [9].

*M. bovis* is a known opportunistic bacterium that can be found normally in the bovine respiratory tract [10]. *M. bovis* becomes pathogenic after stressful situations and initiates clinical signs of BRD, especially in young calves [11]. As other respiratory mycoplasmal pathogens, *M. bovis* colonizes the upper respiratory tract, where it may not cause clinical disease for long periods of time [12]. Under certain host or pathogen factors, disease occurs and results in replication and dissemination to other sites (e.g., lower respiratory tract, middle ear, etc.). Another site for secondary colonization, due to hematologic dissemination from sites of infection, is the joints [9].

Complains by farmers about respiratory signs associated with lameness and joint affections in young calves in Jordan are being reported increasingly and are considered emerging by many field veterinarians. The purpose of this study was to investigate and confirm the etiology of this disease through a reported outbreak of unresponsive pneumonia and arthritis in calves. To the best of our knowledge, this is the first report of such *M. bovis* infection in calves located in Jordan.

## Materials and Methods

### Ethical approval

The study was approved by Jordan University of Science and Technology Animal Care and Use Committee.

### Clinical history of the herd

The farmer and the local veterinarian complained from multiple swollen joints (mainly carpal joints) in a group of young calves that started 2 months before the admission of two calves (one death and one alive) to the Veterinary Health Centre, Jordan University of Science and Technology for postmortem examination. Administration of multiple antibiotic treatments failed to control the infection and swelling of the joints. Within the past 2 months before presentation, the affected calves were moving freely among other peers in the herd. During that period, more cases were reported with the same disease course. The owner had 220 nursing calves within the farm. Of those, 60 calves were affected, and 37 died due to the disease.

### Postmortem examination

**Figure-1:**
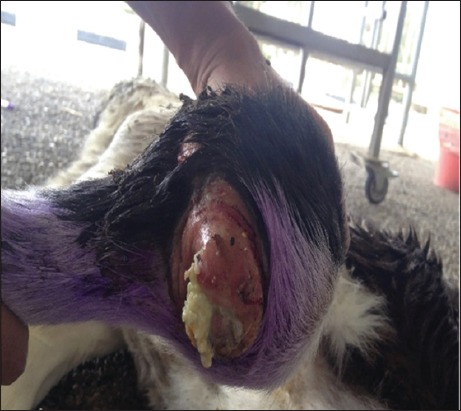
Bovine and left carpal joint. The left carpal joint was markedly swollen, inflamed and discharging a yellowish inspissated caseous material.

**Figure-2:**
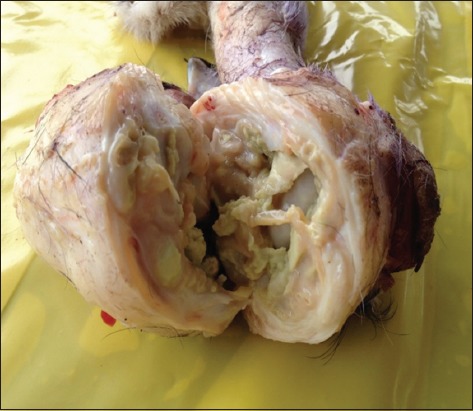
Bovine, calf, and left carpal joint. The joint cavity was filled with yellowish caseous material admixed with variable amounts of fibrins disrupting the normal architecture of the joint. The joint capsule was markedly thickened up to 10× normal.

**Figure-3:**
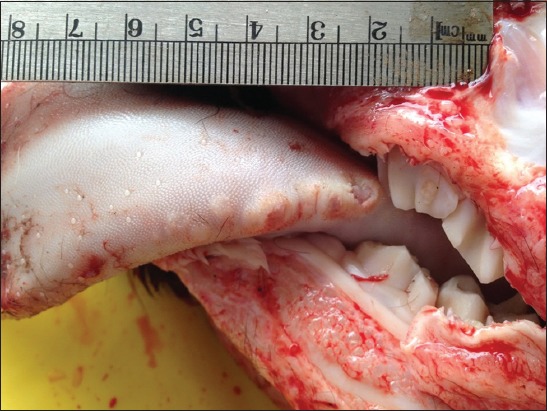
Bovine, calf, and tongue. There were multiple up to 1 cm in diameter erosions and ulcers in the lateral sides of the tongue.

Both calves were in poor nutritional body condition. In both calves, the gross lesions were similar and were restricted to the left carpal joint and the lungs. However, the lesions in the euthanized calf were more severe than the dead one because its left carpal joint was more swollen and inflamed and was discharging a yellowish inspissated caseous material (Figure-1). The joint cavity was filled with yellowish caseous material and admixed with variable amounts of fibrins (Figure-2). These materials were disrupting the normal architecture of articular cartilage causing discoloration, fibrillation, and erosions progressing to the bone necrosis and pathological fracture. The joint capsules and tendon sheaths were markedly thickened up to 10 times the normal size with thick layers of fibrous, fibrinous, and caseous materials. Other joints were within normal limits. In the euthanized calf, there were multiple - approximately 1 cm in diameter - erosions and ulcers in the lateral sides of the tongue (Figure-3).

**Figure-4:**
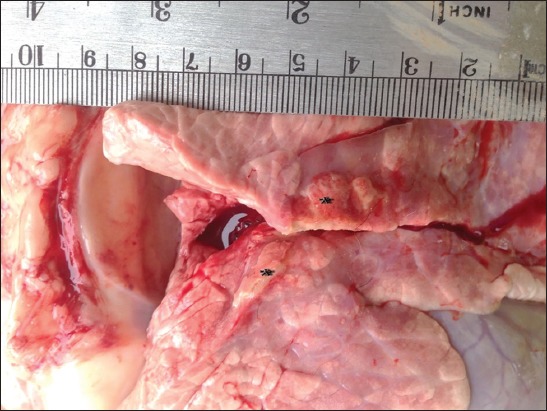
Bovine and lung. Multiple to coalescing yellowish nodules (*) that were elevated above the surrounding pulmonary parenchyma.

More than 60% of the cranioventral lung lobes exhibited gray consolidation with multifocal and coalescing yellowish nodules up to 1.5 cm (Figure-4). These nodules were elevated above the surrounding pulmonary tissue. On the cut surface, these nodules were composed of yellowish caseous materials that were surrounded by a wall of connective tissues. The bronchial and mediastinal lymph nodes were enlarged.

### Bacterial cultures

Lungs and synovial fluids from both calves were submitted for bacterial and mycoplasma cultures. The euthanized calf had not received any antibiotics. The lung tissue and synovial fluid samples were cultured in broth media by inoculating three drops of synovial sample in each 5 ml bijoux bottle containing 3 ml of broth media [7]. A small piece of lung tissue was homogenized using a few drops of PRM broth media in an electrical homogenizer; then, three drops of the homogenate were transferred to a 5 ml bijoux bottle containing 3 ml of broth media. The inoculated media were incubated in a 5% CO_2_ humidified incubator at 37°C. The broths were examined daily for signs of growth, such as a change of pH - indicated by a color change - or a turbidity change in the media. Subculturing from broth onto agar was carried out immediately when growth was evident, or a pH change was seen. The incubation extended to 14 days. The plates were examined under 35× magnification for the presence of a typical “fried egg” appearance of mycoplasma colonies.

### Histopathological examinations

Representative tissue samples from all body organs, including the affected and non-affected organs, were cut and fixed in 10% buffered formalin. After 48 h, the tissues were processed routinely. 4-5 µm thick, sections were made and stained by hematoxylin and eosin stain. A certified pathologist examined the stained tissue sections histologically, and the lesions were recorded.

### RNA and DNA isolation

An RNeasy Mini Kit was used to isolate viral RNA from the spleen and mesenteric lymph nodes. For RNA isolation, briefly, the tissues were homogenized and centrifuged at 3000 rpm for a total of 5 min. Then, 150 µl of the upper phase was moved to a 1.5 ml microcentrifuge tube for the RNA isolation, using the kit. Next, 180 µl of buffer ATL was added to the 1.5 ml microcentrifuge tube, and 20 µl proteinase K was added. Mixed and incubated at 56°C for 10 min, 200 µl of buffer AL was added and mixed; then, 200 µl of absolute ethanol was also added, and the whole mixture was vortexed for 15 s. The whole mixture was then moved to the mini spin column and placed into 2 ml collection tubes, which were centrifuged at 6000× *g* for 1 min. The flow through fluid was discarded. Then, the columns were washed twice using 500 µl of buffers AW1 and AW2. For elution, 75 µl of buffer AE was used to elute the RNA. Isolated RNA was stored at −20°C until analysis by reverse transcription-polymerase chain reaction (RT-PCR).

For DNA extraction, a DNeasy blood and tissue kit from Qiagen, Germany, was used to isolate the genomic DNA from tissue samples, the DNA extraction was performed according to the manufacturer’s protocol.

### PCR amplification

A specific fragment of the 5’ non-translated region of bovine viral diarrhea (BVD) virus genome was amplified according to the published protocol of Givens *et al*. [13]. Hemi-nested RT-PCR was performed using the primers BVD 100 and hepatitis C virus (HCV) 368 in the first round, then BVD 180 and HCV 368 in the second round and according to the conditions mentioned as in Givens *et al*. [13].

For *M. bovis* PCR amplification, a 319 fragment of the *M. bovis* variable surface lipoprotein (Vsp) gene was amplified using the primers Mb1 (5’-AAGGTACACCAGCTAACCCAG-3’) and Mbr2 reverse primer (5’-AATGAAGCTACTGATCCAAG-3’), according to Ghadersohi *et al*. [14]. The PCR amplification was performed using Platinum Taq Mix from Invitrogen, USA. In a total volume of 25 µl, a 2.5 µl was added from the 10× buffer along with 0.75 µl of MgCl_2_, 0.5 µl of dNTPs mix, 1 µl from each primer, 0.1 µl of the Platinum Taq, and 5 µl of the sample DNA. The PCR amplification conditions were an initial 5 min denaturation at 94°C, then 40 cycles with 45 s at 94°C, 45 s at 52°C, and 1 min at 72°C. After cycling, a final extension was applied for 10 min at 72°C. After the amplification, PCR products were run in 2% agarose gel and viewed using an Alpha Innotech Camera.

## Results

**Figure-5:**
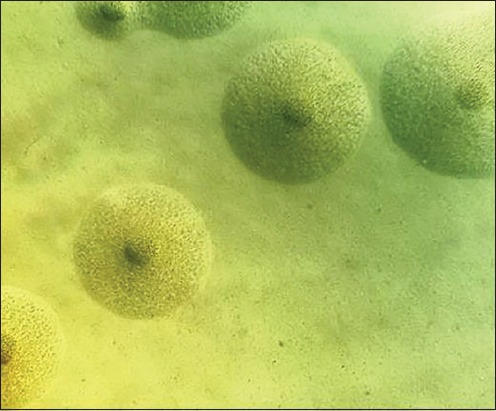
Typical fried egg appearance of the mycoplasma culture PRM agar was isolated from the affected lungs.

Mycoplasmas were isolated from the lung tissue but not from the synovial fluid. The colony morphology on the PRM agar was typically a fried egg appearance, as shown in Figure-5. The isolates grew slowly on the broth and agar media, but after 10 days of incubation, there was a change of color in the broth medium with a distinctive odor.

Pure cultures of *Mannheimia haemolytica* were isolated from the lungs of the calf with no prior treatment. No bacteria were isolated from the treated calf. No other bacteria were isolated from either the lungs or synovial fluids.

Histologically, the yellowish nodule represented multifocal areas of necrogranulomatous pneumonia that was characterized by central areas of necrosis surrounded by a rim of viable and degenerate neutrophils, lymphocytes, plasma cells, and macrophages, which were encased further by multiple layers of fibrous connective tissue. Other pulmonary areas exhibited fibrinosuppurative inflammation. No significant gross or histopathological changes were present elsewhere.

**Figure-6:**
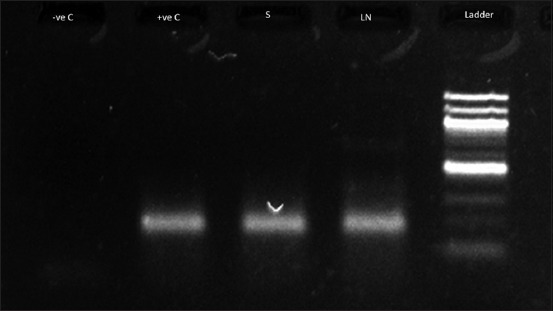
Gel electrophoresis image of the polymerase chain reaction product. –ve C - Is the negative control sample. +ve C - Is the positive control sample. S - Spleen. LN -Mesenteric lymph node. The DNA ladder is 100 bp. The positive band is 213 bp.

**Figure-7:**
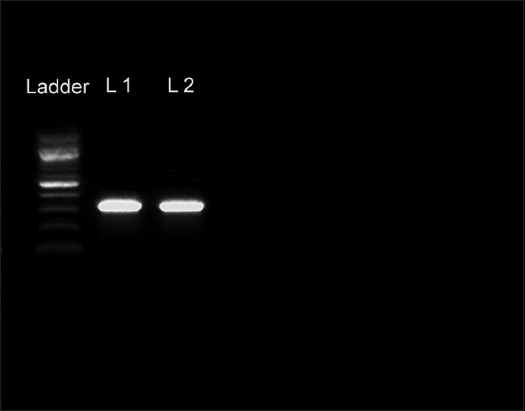
Gel electrophoresis image of the polymerase chain reaction product. L1 - Is the lung from the first calf. L2 - Is the lung from the second calf. The DNA ladder is 100 bp. *Mycoplasma bovis* band is 319 bp.

The bovine tissue RNA samples from mesenteric lymph nodes and spleen in the hemi-nested PCR showed a positive band of 213 bp, comparable to the positive control band of BVD as shown in Figure-6. From the lungs of both calves, a 319 fragment of the *M. bovis* Vsp gene was amplified, as shown in Figure-7.

## Discussion

The history, clinical signs, and pathological findings were indicative of chronic pneumonia and polyarthritis syndrome (CPPS) in the calves. Furthermore, 3 months later, the condition was confirmed for the 2^nd^ time in the same farm. This condition has been recognized widely in feedlot cattle [15].

In this outbreak, *M. bovis* was successfully isolated from the affected lungs that exhibited severe pulmonary lesions. Microbial culture was used traditionally for identification and diagnosis of mycoplasmas [16]. It was reported that *M. bovis* initiates clinical signs of BRD, especially in young calves after stressful situations [11]. Furthermore, *M. bovis* was cultured from the affected lungs in pure culture from naturally infected cattle with CPPS [15]. Moreover, immunohistochemical studies demonstrated the localization of *M. bovis* within the developed lesions, which linked *M. bovis* with the developed lesions [11]. However, it was reported that *M. bovis* could be isolated from cattle without clinical disease or pathological lesions [11]. Moreover, *M. bovis* could be cultured from the majority of feedlot cattle with pneumonia [15]. For these two reasons, bacterial culture was conducted to rule out other possible causes. In addition, pure colonies of *M. haemolytica* were cultured from the lungs in this study. Similar results of coinfection of the pulmonary parenchyma with *M. bovis* and other bacteria were previously reported [15,17].

In this study, the combination of traditional culture and PCR was performed to provide more information about the causative agent. PCR has been reported to have higher efficiency, specificity, and sensitivity than conventional culture-based methods [16]. The secondary bacterial infection might have played an essential role in the severity of the clinical signs as well as the severity of the lesions despite the extensive use of several antibiotics was used to treat those animals [18]. It was reported that many strains of *M. bovis* are resistant to the antibiotic treatments, as occurred in this outbreak [8,10]. This failure of antimicrobial therapy might also suggest the presence of more than one pathogen that was responsible for the unresponsive treatment [8,19-22]. Furthermore, *in vitro* studies in Europe have reported high minimum inhibitory concentrations against circulating *M. bovis* for many of the commercially available antimicrobials [21].

A predisposing viral infection was suspected to exacerbate the clinical course of the disease in this outbreak. Indeed, multiple, variably sized ulcers (up to 1 cm in diameter) were found in the lateral sides of the tongue in one calf in this study, which suggested a possible BVDV infection. Different studies reported an association between BVDV infection and *M. bovis* [15,23]. It was found that BVDV infection was more common in pneumonic calves of bacterial etiology than in those dying of other causes [15].

*M. bovis* infection is characterized by distinctive gross pathological lesions within the affected lungs similar to those lesions that were found in our case in this study [6]. These lesions are consistent of cranioventral distribution and multiple, variably sized caseous abscesses. Similar pulmonary lesions have been experimentally induced after infecting calves with *M. bovis* [6]. In this outbreak, the lesions were found in the lungs and joints. Similar findings were reported by different studies where the pulmonary lesions were accompanied with polyarthritis [24,25].

In this outbreak, the infection has emerged in and remained limited to the calves. No clinical abnormalities were noted in the adult animals. Similar findings were previously reported in one outbreak of *M. bovis* infection in calves [26]. It is believed that the introduction of an asymptomatic carrier with no apparent clinical signs to the herd initiated the horizontal transmission of the disease. Furthermore, another important mean of mycoplasma transmission to calves is the ingestion of milk contaminated with mycoplasma obtained from a cow affected with mycoplasma mastitis [11].

## Conclusion

To the best of our knowledge, the emergence of chronic unresponsive *M. bovis* arthritis and pneumonia has not previously been documented in Jordan. Furthermore, multiple infectious agents could be encountered in veterinary practices.

## Authors’ Contributions

WMH and WMA had carried out the design and data processing. WMH carried the pathology section. WMA carried the bacterial and mycoplasma culture. MMA designed and carried out the molecular section. SMA carried out the clinical section. All authors drafted the final manuscript. The final manuscript was read and approved by all authors.
